# General Hospitals

**Published:** 1894-12-22

**Authors:** 


### SWEET CHARITY'S GUIDE FOR CHRISTMAS GIVERS.

### Being the Christmas Number of "The Hospital."

### GENERAL HOSPITALS.

**Charing Cross Hospital**, Agar Street, West Strand. —This institution is situated in the midst of some of the most crowded thoroughfares in the metropolis, and has therefore to provide for a greater number of accidents, *i.e.*, urgent cases, than probably any other hospital of its size. All such cases are immediately admitted without delay or difficulty. About 23,500 patients were relieved during the past year, more than two-thirds of which were cases of accident or emergency. Can any stronger appeal be made than this to the many visitors who usually come to London, or to the public generally? We think not. Yet, the need for pecuniary help is great and pressing, and the Council earnestly appeal for donations towards meeting the deficiency and for annual subscriptions. The present debt amounts to £11,000, and there are no investments which can be realised to relieve the hospital of this heavy burden. Secretary, Mr. A. E. Reade; Matron, Miss H. Gordon.

**German Hospital**, Dalston, N.E.—This hospital contains twenty beds, and admits all who are conversant with the German language, without distinction of nationality or creed, as well as accident and emergency cases. During last year 1,457 in-patients, and 19,324 out-patients were treated at the Hospital Dispensary, and a further 6,423 at the Eastern and Western Dispensaries. Six beds are set aside for patients paying one and a-half to two guineas a week. The convalescent home also has proved most beneficial. The annual expenditure amounts to about £9,800, the more reliable income to £5,100, leaving the sum of £4,700 to be raised by special appeal. Superintendent and Secretary, Mr. H. Gülich; Matron, Miss Christiane Bürger.

**Great Northern Central Hospital**, Holloway Road, N.—This hospital is entirely free to the sick poor, no letters of recommendation being required to gain admission. It was founded at King's Cross in 1856, but was removed to Holloway in 1887. The new hospital contains 155 beds, and was completed in April last. It is one of the most beautiful and perfect in London, and every detail has been carried out in accordance with modern scientific knowledge. Over 1,200 in-patients and 26,000 out-patients are treated annually, but the increased number of beds now available will nearly double the number of in-patients. The hospital is unendowed, and the reliable income quite inadequate to meet the necessary expenditure—over £8,000 being required annually from voluntary sources. There is also a debt of £12,000 on the building fund—a heavy burden, hampering the good work of the hospital. Annual subscriptions and donations may be sent to the secretary, Lewis H. Glenton-Kerr.

**Guy's Hospital**, Borough, S.E.—Guy's is too old a friend of the public to need much to be said in its favour; but so long as it has closed wards and is crippled by poverty it is a reproach to the metropolis. The nett income of the hospital derived from its landed estates remains at £20,000 per annum less than it was before the agricultural depression, with no present prospect of improvement. There is left, therefore, only an assured income of £21,000 to maintain the 500 beds now open, which cannot be kept up at a less cost than £38,000 per annum. The greater part of this deficiency can only be met in the future as in the past, by liberal contributions from the public exclusive of any further aid from the same source, required to re-open 100 beds still remaining vacant, for which there is a pressing demand. Matron, Miss Nott Bower; Superintendent, Dr. Perry.

**King's College Hospital**, Portugal Street, W.C.—That this hospital has much claim on the public for the good work it does goes without saying. Its finances are not, however, in that flourishing condition that they ought to be in, were the hospital supported as it should be. First, there is a need for money to meet the current expenses, as of assured income it has but £1,000 to meet an expenditure of £19,000. Next comes the need of subscriptions towards paying off a loan of £2,000, due to the bankers; and thirdly, towards a Special Five Years' Maintenance Fund of £50,000 which, was started by a meeting held at Grosvenor House under the presidency of His Grace the Duke of Westminster, K.G. The sum at present given and promised amounts to about £9,000. The average annual receipts of the hospital during the past ten years have been about £16,500, including legacies, but the annual expenditure is over £19,000. We must draw special attention to the convalescent home, which secures a period of ease for the patients sent to it from the hospital wards. As considerably over 24,000 patients are yearly admitted to the benefits of the hospital, it should surely have brought its claims for aid to the doors of many of the giving public, who, now knowing its needs, will, we feel confident, do their utmost to assist it. Secretary, Rev. N. Bromley; Sister-Matron, Miss Monk.

**London Hospital**, Whitechapel Road, E.—This is the greatest hospital in this country. From this fact, and from the fact that it has been persistently attacked by a small clique, in spite of their contentions having been shown to be ill-founded over and over again, we make no doubt that the public will give largely to its funds, in testimony of their appreciation of the enormous value of the work which it does for the poor of East London. It includes special departments under eminent medical men for the treatment of all classes of disease, the number of beds devoted to children being greater than those to be met with in most children's hospitals. The character of the work done and its value to the public may be realised from the statement that the able Matron, Miss Lückes, has three assistants under her, in addition to two night superintendents, 19 day sisters, and 220 staff and probationer nurses. This great hospital of the East-end is in serious want of funds, as the committee depend on voluntary contributions for £40,000 a year to enable them to maintain the 640 beds which are daily occupied by urgent cases. House Governor and Secretary, Mr. G. Q. Roberts; Matron, Miss Eva C Lückes.

**London Homœopathic Hospital**, Great Ormond Street, W.C.—This hospital has a well-earned reputation for efficiency and careful management. £10,000 will be required to complete the new building, now in progress at a cost of £45,000, of which the sum of £35,000 has already been contributed. The demand for the nurses trained in the hospital continues to increase. Connected with the hospital is the Homœopathic Convalescent Home, 36, Enys Road, Eastbourne. This gives to women and children who have recovered from illness the benefits of a temporary Eastbourne home, with good food and careful attention. Patients are received from all parts, as the home is not restricted to those who have been in the hospital, and adults contribute 7s. per week, children 3s. 6d. per week. Secretary, G. A. Cross.

**Metropolitan Hospital**, Kingsland Road, N.E.—Situated in the heart of the poor and densely populated districts of Shoreditch, Haggerston, Hackney, Bethnal Green, Hoxton, Dalston, and De Beauvoir Town, the hospit receives a very large number of accidents and casualties of all kinds, and the demands made on its resources, medical and surgical, are incessant and urgent. That the hospital is needed in it present position is shown by the fact that both the in and out patiests have been steadily on the increase for several years past, the numbers treated last year being, in-patients, 774; out-patients, attendances, 73,283. In spite of this good work the expenditure last year exceeded the income by £2,062, leaving a debt of over £4,000 incurred during the past three years. It is deplorable that the work of the charity should be crippled for lack of funds, owing to which several beds have had to be closed, leaving only 54 now available for in-patients, although there is accommodation for 160. Many urgent cases have therefore constantly to be refused admission, and unless money is immediately forthcoming it will be necessary to still further reduce the number of beds. We are sure that the public will readily respond to the committee's urgent appeal for help, and contributions should be forwarded to the Secretary, Mr. C. H. Byers, at the Hospital, or to the Bankers, Messrs. Glyn, Mills, and Co., 67, Lombard Street, E.C.

**The Middlesex Hospital**, Mortimer Street, W.—This institution does a large share in the beneficent work of relief carried on so courageously by our voluntary hospitals, despite all the difficulties in their way. This is at once apparent when we say that the hospital contains 321 beds; that the in-patients for last year numbered 2,513, and the out-patients 30,252 (total, 32,765); that the income from all sources, including legacies, was £20,357, and the expenditure £37,184; deficiency, £16,827. Last year the hospital was closed for three months to enable extensive repairs and sanitary alterations, necessitated by the age of the building, to be carried out. The expense of the works executed was nearly £8,000, and the board earnestly appeal for aid in meeting this large expenditure which has been made solely in the interests of the patients. The cancer wards are a distinguishing feature of the hospital, 35 beds being specially set aside for these cases, and the extra nursing, costly treatment, and unlimited dietary accorded to the sufferers from this terrible disease add largely to the expenses of the hospital. Secretary Superintendent, Mr. F. Clare Melhado; Lady Superintendent, Miss Thorold.

**Miller Hospital and Royal Kent Dispensary**, Greenwich Road, S.E. Established 1783.—Opened in 1885 as a hospital in memory of the late Canon Miller, D.D.; this was the first hospital built in England with circular wards. During the year 15,000 cases were attended at the hospital, which is entirely dependent on voluntary contributions. About £800 more is required to carry on the work. Secretary, Mr. James Marks; Matron, Miss E. L. Underhill; Hon. Secretary, Major-General G. R. Roberts.

**North-West London Hospital**, Kentish Town Road, was founded in 1878, and is the only institution of the kind in the north-west district. It has 53 beds, of which 18 are for sick children. Last year there were 512 in-patients and 44,506 attendances as out-patients. A noticeable feature in the statement of accounts is the comparatively small item for salaries of officers and other costs of management. Its admirably appointed wards are worth a visit, and the committee do well in cordially inviting visitors to see and judge for themselves. There is also a Samaritan Fund, and a soup kitchen (maintained by the Treasurer, Mr. George Herring), where about 2,000 dinners weekly are being distributed. The annual expenditure is never under £4,000, towards which the annual subscriptions yield less than £900. A large deficiency has therefore to be provided for by special benefactions. The committee very earnestly plead for assistance. Secretary, Mr. Alfred Craske.

**Royal Free Hospital**, Gray's Inn Road, W.C.—About £6,000 is now required. From the ruinous condition of the old front building, which was formerly a barrack for the Light Horse Volunteers, it became absolutely necessary to rebuild this portion of the hospital. To carry out this work, which will provide much-needed isolation wards, casualty-rooms, and dispensary, and to provide a new steam laundry, the help of those who have the means towards supplying the amount now needed is very earnestly solicited, and also to increase the current revenue by new annual subscriptions. The Royal Free Hospital was the first general hospital in London to open its doors freely to the sick and suffering poor without letters of recommendation from the governors. For 67 years it has adhered to this excellent system, and during that period over two millions of poor people have received within its walls the best medical and surgical advice and treatment. The hospital now opens its doors to lady students. Secretary, Mr. Conrad W. Thies.

**St. George's Hospital**, Hyde Park Corner, S.W.—Occupying a commanding position, there is reason to believe that it suffers in consequence, and we hope that all who pass St. George's Hospital in future will contribute something to its funds in the course of the year. Secretary and Superintendent, Mr. C. L. Todd; Matron, Mrs. Coster.

**St. Luke's Hospital**, London, E.C.—This hospital continues its work of perfecting its building and adding to the comfort of its inmates. The expenses are necessarily very heavy, and the income quite inadequate to meet them. A convalescent establishment in connection with the hospital is now open at St. Lawrence, near Ramsgate; and, in view of the good work that has already been done through this medium, the committee are encouraged to confidently appeal for help to this one of the oldest of the London charities. Treasurer, Edward W. Nix, Esq.; Secretary, Percy de Bathe, M.A.

**Seamen's Hospital Society, "Dreadnought,"** Greenwich, S.E.—This institution, which since the year 1821 has been the only charity that has devoted itself to the relief and amelioration of the sufferings of sick seamen in the Port of London, has been doing an even larger amount of work in the year that is drawing to a close than in any previous twelve months. The society has so far extended its operations that it now consists of the "Dreadnought" Hospital ashore at Greenwich, another hospital in the Victoria and Albert Dock, a dispensary in the East India Dock Road, and another at Gravesend. Last year in these various establishments over 18,000 sick, injured, and shipwrecked sailors were treated. The British public owe a special debt of gratitude to the sailor, and of all men, when sick, the sailor is the most in need of succour and assistance. It is to the Seamen's Hospital Society that a sailor looks in the hour of his need, and the committee therefore urgently appeal to the public to help. Funds are urgently needed, so that the expenditure may not again exceed the income for the current year. Secretary, Mr. P. Michelli; Matron, Miss Cooke.

**University College Hospital**, Gower Street, W.—The North London or University College Hospital is mainly dependent upon voluntary contributions, the income on which it can rely being only £6,000, whilst the necessary annual expenditure is very nearly £20,000, and the present debt exceeds £10,000. The rebuilding of the hospital, too, upon a more modern plan, as soon as possible, is an urgent necessity, and for this purpose a fund was instituted in 1884, named the "Jubilee Endowment and Building" Fund, to which contributions are earnestly invited. Secretary, Mr. Newton H. Nixon.

**Westminster Hospital**, Broad Sanctuary, S.W.—Out of an expenditure of £15,000, less than £3,000 is assured to this charity, so that about £12,000 has to be made up each year in subscriptions. The expenditure for the year just about to close is more than £5,000 in excess of the receipts. The hospital does an immense service to the country by training a large number of excellent nurses. Secretary, Mr. Sidney M. Quennell; Matron, Miss Pyne.

**West London Hospital**, Hammersmith Road, W. (101 beds), is the nearest for a population of nearly 500,000 persons. The accommodation for in-patients is lamentably short of the number requiring admission, and in each succeeding year the fact is brought more to the front than in its predecessor. Last year (1893), the beds were occupied by 1,611 patients, and the out-patient department was attended by 31,446 others. Its endowment yields an annual income of only £115 and it is greatly to be feared that the income of the year now closing will be considerably short of the necessary expenditure for maintenance. The Board appeal for funds for continuing the erection of a section of the plan of extension to accommodate about 80 beds, as well as for carrying on the present work. Secretary, Mr. R. J. Gilbert; Lady Superintendent, Miss Hardy.

